# Supplementary material for: Evidence to Support Inclusion of Pharmacogenetic Biomarkers in Randomised Controlled Trials
Source: J Pers Med. 2019 Sep 1;9(3):42. doi: 10.3390/jpm9030042 (PMC6789450; doi:10.3390/jpm9030042)
Supplement: Supplementary file 1 [file jpm-09-00042-s001.pdf]

**TARGET (2005)[1,2]**				
Reference No.	Type of reference	Authors	Year	DOI
1	Observational – cohort	Dubinsky et al [3]	2000	10.1016/S0016-5085(00)70140-5
2	Observational – cohort	Weinshilboum and Sladek [4]	1980	n/a
3	Observational – case control	Lennard et al [5]	1989	10.1038/clpt.1989.119
4	Observational – cohort	McLeod et al [6]	1994	10.1038/clpt.1994.4
5	Observational – cohort	Yates et al [7]	1997	10.7326/0003-4819-126-8-199704150-00003
6	Guidelines	British Society of Rheumatology [not found]	2000	n/a
7	Observational – of assay use	Holme et al [8]	2002	10.1093/qjmed/95.7.439
8	Observational – cohort	Bloomfeld & Onken [9]	2003	10.1046/j.1365-2036.2003.01392.x
9	Observational – cohort	McLeod et al [10]	1999	10.1046/j.1365-2141.1999.01416.x
10	Observational – cohort	Black et al [11]	1998	10.7326/0003-4819-129-9-199811010-00007
11	Observational – cohort	Pandya et al [12]	2002	10.1016/S0041-1345(02)02963-9
12	Expert opinion	Seidman [13]	2003	n/a
13	Observational – cohort	Murphy & Atherton [14]	2002	10.1046/j.1365-2133.2002.04922.x
14	Systematic review	Phillips et al [15]	2001	10.1001/jama.286.18.2270
15	Case study	Tavadia et al [16]	2000	10.1067/mjd.2000.103980
16	Cost-effectiveness study	Marra et al [17]	2002	n/a
17	Qualitative work	Tan et al [18]	1997	10.1046/j.1365-2133.1997.d01-1198.x

**EU-PACT (2009)[19]**				
Reference No.	Type of reference	Authors	Year	DOI
1	Editorial	Rosendaal [20]	1996	10.1056/NEJM199608223350810
2	Observational – retrospective cohort	James et al [21]	1992	n/a
3	Observational – cohort	Penning-van Beest et al [22]	2001	n/a
4	Observational – case control	Hylek et al [23]	1996	10.1056/NEJM199608223350802

5	Editorial	Pirmohamed [24]	2006	10.1111/j.1365-2125.2006.02806.x
6	Observational – case control	Penning-van Beest et al [25]	2002	10.1016/S0895-4356(01)00485-1
7	Observational – cohort	Carlquist et al [26]	2006	10.1007/s11239-006-9030-7
8	Observational – cohort	Schalekamp et al [27]	2007	10.1007/s00228-007-0268-6
9	Observational – case control	Schalekamp et al [28]	2008	10.1001/archinternmed.2007.32
10	Observational – cohort	Gage et al [29]	2004	10.1160/TH03-06-0379
11	Observational – cohort (healthy volunteers)	Bodin et al [30]	2005	10.1182/blood-2005-01-0341
12	Observational – cohort	Wadelius et al [31]	2009	10.1182/blood-2008-04-149070
13	Observational – cohort	Schalekamp et al [32]	2006	10.1016/j.clpt.2006.04.006
14	Observational – cohort	Schalekamp et al [33]	2007	10.1038/sj.clpt.6100036
15	Observational – case control	Reitsma et al [34]	2005	10.1371/journal.pmed.0020312
16	Observational – cohort	Wadelius et al [35]	2005	10.1038/sj.tpj.6500313
17	Observational – cohort	D'Andrea et al [36]	2005	10.1182/blood-2004-06-2111
18	Observational – cohort (GWAS)	Takeuchi et al [37]	2009	10.1371/journal.pgen.1000433
19	Observational – cohort	Caldwell et al [38]	2008	10.1182/blood-2007-11-122010
20	Observational – cohort	Schelleman et al [39]	2008	10.1038/clpt.2008.101
21	Observational – cohort	Klein et al [40]	2009	10.1056/NEJMoa0809329.
22	Observational – cohort	Perini et al [41]	2008	10.1038/clpt.2008.166
23	Observational – cohort	Sconce et al [42]	2005	10.1182/blood-2005-03-1108
24	Observational – cohort	Tham et al [43]	2006	10.1016/j.clpt.2006.06.009
25	Observational – cohort	Gage et al [44]	2008	10.1038/clpt.2008.10
26	Cost-effectiveness analysis	Eckman et al [45]	2009	10.7326/0003-4819-150-2-200901200-00005
27	Cost-effectiveness analysis	Schalekamp et al [46]	2006	10.1016/j.clpt.2006.03.008
28	Literature review	Hughes and Pirmohamed [47]	2007	10.2165/00019053-200725110-00001

**SHIVA (2014)[48]**				
Reference No.	Type of reference	Authors	Year	DOI
1	Randomised controlled trial	Slamon et al [49]	2001	10.1056/NEJM200103153441101
2	Observational – cohort	Lièvre et al [50]	2008	10.1200/JCO.2007.12.5906
3	Case study	Joensuu et al [51]	2001	10.1056/NEJM200104053441404
4	Literature review	DiMasi & Grabowski [52]	2007	10.1200/JCO.2006.09.0803
5	Literature review	Von Hoff [53]	1998	n/a
6	Randomised controlled trial	Thatcher et al [54]	2005	10.1016/S0140-6736(05)67625-8

7	Randomised controlled trial	Mok et al [55]	2009	10.1056/NEJMoa0810699
8	Observational – cohort	Von Hoff et al [56]	2010	10.1200/JCO.2009.26.5983
9	Editorial	Doroshow [57]	2010	10.1200/JCO.2010.31.1472
10	Randomised controlled trial	Kim et al [58]	2011	10.1158/2159-8274.CD-10-0010

**GGST statin trial (2016)[59]**				
Reference No.	Type of reference	Author(s)	Year	DOI
1	Epidemiology – American Heart Association	Mozaffarian et al [60]	2015	10.1161/CIR.0000000000000157
2	Editorial	Greenland and Lauer [61]	2015	10.1001/jama.2015.7434
3	Meta-analysis	Cholesterol Treatment Triallists' Collaborators [62]	2012	10.1016/S0140-6736(12)60367-5
4	Meta-analysis	Cholesterol Treatment Triallists' Collaborators [63]	2015	10.1016/S0140-6736(14)61368-4
5	Cochrane review	Taylor et al [64]	2013	10.1002/14651858.CD004816.pub5
6	Guidelines - American College of Cardiology/American Heart Association Task Force	Stone et al [65]	2013	10.1016/j.jacc.2013.11.002
7	Observational – cohort	Pencina et al [66]	2014	10.1056/NEJMoa1315665
8	Literature review	Hirsh et al [67]	2015	10.1016/j.jacc.2015.05.030
9	Observational – cohort	Bermingham et al [68]	2011	10.1016/j.clinthera.2011.07.007
10	Observational – cohort	Ho et al [69]	2008	10.1016/j.ahj.2007.12.011
11	Observational – cohort	Vodonos et al [70]	2015	10.1016/j.ejim.2015.02.014
12	Observational – cohort	Franklin et al [71]	2015	10.1002/pds.3787
13	Systematic review	De Vera et al [72]	2014	10.1111/bcp.12339
14	Literature review	Osterburg and Blaschke [73]	2005	10.1056/NEJMra050100
15	Qualitative study	Fung et al [74]	2010	n/a
16	Qualitative study	Cohen et al [75]	2012	10.1016/j.jacl.2012.03.003
17	Literature review	Ong et al [76]	2012	10.2217/pgs.12.2
18	Guidelines – American College of Cardiology/American Heart Association/National Heart, Lung and Blood Institute	Pasternak et al [77]	2002	10.1016/S0735-1097(02)02030-2
19	Expert opinion	Thompson et al [78]	2006	10.1016/j.amjcard.2005.12.013
20	Literature review/expert opinion	Alfirevic et al [79]	2014	10.1038/clpt.2014.121

21	Literature review	Patel et al [80]	2015	10.1016/j.atherosclerosis.2015.03.025
22	Guidelines – European Atherosclerosis Society	Stroes et al [81]	2015	10.1093/eurheartj/ehv043
23	Observational – case control	Link et al [82]	2008	10.1056/NEJMoa0801936
24	Meta-analysis	Hou et al [83]	2015	10.1097/MD.0000000000001268
25	Observational – cohort	Pasanen et al [84]	2006	10.1007/s00228-006-0123-1
26	In vitro work	Kimoto et al [85]	2012	10.1021/mp300379q
27	Randomised controlled trial	Voora et al [86]	2009	10.1016/j.jacc.2009.04.053
28	Guidelines – Clinical Pharmacogenetics Implementation Consortium	Wilke et al [87]	2012	10.1038/clpt.2012.57
29	Observational – cohort	Birmingham et al [88]	2015	10.1007/s00228-014-1801-z
30	Observational – cohort/meta-analysis	de Keyser et al [89]	2014	10.1097/FPC.0000000000000018
31	Sub-study of larger randomised controlled trial	Danik et al [90]	2013	10.1016/j.ahj.2013.01.025
32	Sub-study of larger randomised controlled trial	Martin et al [91]	2011	10.1111/j.1365-2125.2011.04090.x
33	Literature review	Niemi et al [92]	2011	10.1124/pr.110.002857
34	Guidelines – Clinical Pharmacogenetics Implementation Consortium	Ramsey et al [93]	2014	10.1038/clpt.2014.125
35	Literature review	Voora and Ginsburg [94]	2012	10.1016/j.jacc.2012.01.067
36	Observational – cohort	Donnelly et al [95]	2011	10.1038/clpt.2010.255
37	Observational – cohort (pilot study)	Li et al [96]	2014	10.3390/jpm4020147

NCT02664350 (2018)[97]

Reference No.	Type of reference	Author(s)	Year	DOI
1	Guidelines – National Cancer Institute	PDQ Supportive and Palliative Care Editorial Board [98]	2002	n/a
2	Guidelines – National Comprehensive Cancer Network	National Comprehensive Cancer Network [99]	2017	n/a
3	Randomised controlled trial	Temel et al [100]	2010	10.1056/NEJMoa1000678
4	Guidelines – European Association for Palliative Care	Caraceni et al [101]	2012	10.1016/S1470-2045(12)70040-2
5	Expert panel	Fine et al [102]	2009	10.1016/j.jpainsymman.2009.06.002
6	Observational – cohort	Zhao et al [103]	2014	10.1200/JCO.2013.50.6071
7	Randomised controlled trial (in twins)	Angst et al [104]	2012	10.1016/j.pain.2012.02.022
8	Literature review	Fillingim et al [105]	2008	10.1111/j.1601-0825.2008.01458.x

9	Observational – cohort	Gan et al [106]	2007	10.1007/BF03256239
10	Case study	Susce et al [107]	2006	10.1016/j.pnpbp.2006.03.018
11	Guidelines – Clinical Pharmacogenetics Implementation Consortium	Crews et al [108]	2014	10.1038/clpt.2013.254
12	Observational – cohort	Baber et al [109]	2015	10.1038/tpj.2015.3
13	Case study	Ciszkowski et al [110]	2009	10.1056/NEJMc0904266
14	Case study	Gasche et al [111]	2004	10.1056/NEJMoa041888
15	Randomised controlled trial	Eckhardt et al [112]	1998	10.1016/S0304-3959(98)00021-9
16	Randomised controlled trial	Lötsch et al [113]	2009	10.1016/j.pain.2009.03.023
17	Observational – cohort	Andreassen et al [114]	2012	10.1007/s00228-011-1093-5
18	Randomised controlled trial	Samer et al [115]	2010	10.1111/j.1476-5381.2010.00673.x
19	Randomised controlled trial	Zwisler et al [116]	2009	10.1111/j.1742-7843.2009.00378.x
20	Observational – cohort	Zwisler et al [117]	2010	10.1111/j.1399-6576.2009.02104.x

1. Newman, W.G.; Payne, K.; Tricker, K.; Roberts, S.A.; Fargher, E.; Pushpakom, S.; Alder, J.E.; Sidgwick, G.P.; Payne, D.; Elliott, R.A., *et al.* A pragmatic randomized controlled trial of thiopurine methyltransferase genotyping prior to azathioprine treatment: the TARGET study. *Pharmacogenomics* **2011**, *12*, 815-826.
2. Ollier, B.; Newman, B.; Payne, K.; Poulton, K.; Andrews, J.; Elliott, R.; Ray, D.; Elles, R.; Houston, B.; Bruce, I., *et al.* The TARGET Study TPMT: AZA Response to Genotyping and Enzyme Testing Protocol: A prospective randomised controlled trial of thiopurine methyltransferase (TPMT) genotyping in the management of patients, prior to commencement of AZA treatment. University of Manchester, Ed. 2005.
3. Dubinsky, M.C.; Lamothe, S.; Yang, H.Y.; Targan, S.R.; Sinnett, D.; Theoret, Y.; Seidman, E.G. Pharmacogenomics and metabolite measurement for 6-mercaptopurine therapy in inflammatory bowel disease. *Gastroenterology* **2000**, *118*, 705-713.
4. Weinshilboum, R.M.; Sladek, S.L. Mercaptopurine pharmacogenetics: monogenic inheritance of erythrocyte thiopurine methyltransferase activity. *American journal of human genetics* **1980**, *32*, 651-662.
5. Lennard, L.; Van Loon, J.A.; Weinshilboum, R.M. Pharmacogenetics of acute azathioprine toxicity: relationship to thiopurine methyltransferase genetic polymorphism. *Clinical pharmacology and therapeutics* **1989**, *46*, 149-154.
6. McLeod, H.L.; Lin, J.S.; Scott, E.P.; Pui, C.H.; Evans, W.E. Thiopurine methyltransferase activity in American white subjects and black subjects. *Clinical pharmacology and therapeutics* **1994**, *55*, 15-20.
7. Yates, C.R.; Krynetski, E.Y.; Loennechen, T.; Fessing, M.Y.; Tai, H.L.; Pui, C.H.; Relling, M.V.; Evans, W.E. Molecular diagnosis of thiopurine S-methyltransferase deficiency: genetic basis for azathioprine and mercaptopurine intolerance. *Annals of internal medicine* **1997**, *126*, 608-614.

8. Holme, S.A.; Duley, J.A.; Sanderson, J.; Routledge, P.A.; Anstey, A.V. Erythrocyte thiopurine methyl transferase assessment prior to azathioprine use in the UK. *QJM : monthly journal of the Association of Physicians* **2002**, *95*, 439-444.
9. Bloomfeld, R.S.; Onken, J.E. Mercaptopurine metabolite results in clinical gastroenterology practice. *Alimentary pharmacology & therapeutics* **2003**, *17*, 69-73.
10. McLeod, H.L.; Coulthard, S.; Thomas, A.E.; Pritchard, S.C.; King, D.J.; Richards, S.M.; Eden, O.B.; Hall, A.G.; Gibson, B.E. Analysis of thiopurine methyltransferase variant alleles in childhood acute lymphoblastic leukaemia. *British journal of haematology* **1999**, *105*, 696-700.
11. Black, A.J.; McLeod, H.L.; Capell, H.A.; Powrie, R.H.; Matowe, L.K.; Pritchard, S.C.; Collie-Duguid, E.S.; Reid, D.M. Thiopurine methyltransferase genotype predicts therapy-limiting severe toxicity from azathioprine. *Annals of internal medicine* **1998**, *129*, 716-718.
12. Pandya, B.; Thomson, W.; Poulton, K.; Bruce, I.; Payne, D.; Qasim, F. Azathioprine toxicity and thiopurine methyltransferase genotype in renal transplant patients. *Transplant Proc* **2002**, *34*, 1642-1645.
13. Seidman, E.G. Clinical use and practical application of TPMT enzyme and 6-mercaptopurine metabolite monitoring in IBD. *Reviews in gastroenterological disorders* **2003**, *3 Suppl 1*, S30-38.
14. Murphy, L.A.; Atherton, D. A retrospective evaluation of azathioprine in severe childhood atopic eczema, using thiopurine methyltransferase levels to exclude patients at high risk of myelosuppression. *The British journal of dermatology* **2002**, *147*, 308-315.
15. Phillips, K.A.; Veenstra, D.L.; Oren, E.; Lee, J.K.; Sadee, W. Potential role of pharmacogenomics in reducing adverse drug reactions: a systematic review. *JAMA* **2001**, *286*, 2270-2279.
16. Tavadia, S.M.; Mydlarski, P.R.; Reis, M.D.; Mittmann, N.; Pinkerton, P.H.; Shear, N.; Sauder, D.N. Screening for azathioprine toxicity: a pharmacoeconomic analysis based on a target case. *Journal of the American Academy of Dermatology* **2000**, *42*, 628-632.
17. Marra, C.A.; Esdaile, J.M.; Anis, A.H. Practical pharmacogenetics: the cost effectiveness of screening for thiopurine s-methyltransferase polymorphisms in patients with rheumatological conditions treated with azathioprine. *The Journal of rheumatology* **2002**, *29*, 2507-2512.
18. Tan, B.B.; Lear, J.T.; Gawkrodger, D.J.; English, J.S. Azathioprine in dermatology: a survey of current practice in the U.K. *The British journal of dermatology* **1997**, *136*, 351-355.
19. van Schie, R.M.; Wadelius, M.I.; Kamali, F.; Daly, A.K.; Manolopoulos, V.G.; de Boer, A.; Barallon, R.; Verhoef, T.I.; Kirchheiner, J.; Haschke-Becher, E.*, et al.* Genotype-guided dosing of coumarin derivatives: the European pharmacogenetics of anticoagulant therapy (EU-PACT) trial design. *Pharmacogenomics* **2009**, *10*, 1687-1695.
20. Rosendaal, F.R. The Scylla and Charybdis of oral anticoagulant treatment. *The New England journal of medicine* **1996**, *335*, 587-589.
21. James, A.H.; Britt, R.P.; Raskino, C.L.; Thompson, S.G. Factors affecting the maintenance dose of warfarin. *J Clin Pathol* **1992**, *45*, 704-706.
22. Penning-van Beest, F.J.; van Meegen, E.; Rosendaal, F.R.; Stricker, B.H. Characteristics of anticoagulant therapy and comorbidity related to overanticoagulation. *Thromb Haemost* **2001**, *86*, 569-574.
23. Hylek, E.M.; Skates, S.J.; Sheehan, M.A.; Singer, D.E. An analysis of the lowest effective intensity of prophylactic anticoagulation for patients with nonrheumatic atrial fibrillation. *The New England journal of medicine* **1996**, *335*, 540-546.
24. Pirmohamed, M. Warfarin: almost 60 years old and still causing problems. *British journal of clinical pharmacology* **2006**, *62*, 509-511.

25. Penning-van Beest, F.J.; Geleijnse, J.M.; van Meegen, E.; Vermeer, C.; Rosendaal, F.R.; Stricker, B.H. Lifestyle and diet as risk factors for overanticoagulation. *Journal of clinical epidemiology* **2002**, *55*, 411-417.
26. Carlquist, J.F.; Horne, B.D.; Muhlestein, J.B.; Lappe, D.L.; Whiting, B.M.; Kolek, M.J.; Clarke, J.L.; James, B.C.; Anderson, J.L. Genotypes of the cytochrome p450 isoform, CYP2C9, and the vitamin K epoxide reductase complex subunit 1 conjointly determine stable warfarin dose: a prospective study. *Journal of thrombosis and thrombolysis* **2006**, *22*, 191-197.
27. Schalekamp, T.; van Geest-Daalderop, J.H.; Kramer, M.H.; van Holten-Verzantvoort, A.T.; de Boer, A. Coumarin anticoagulants and co-trimoxazole: avoid the combination rather than manage the interaction. *Eur J Clin Pharmacol* **2007**, *63*, 335-343.
28. Schalekamp, T.; Klungel, O.H.; Souverein, P.C.; de Boer, A. Increased bleeding risk with concurrent use of selective serotonin reuptake inhibitors and coumarins. *Archives of internal medicine* **2008**, *168*, 180-185.
29. Gage, B.F.; Eby, C.; Milligan, P.E.; Banet, G.A.; Duncan, J.R.; McLeod, H.L. Use of pharmacogenetics and clinical factors to predict the maintenance dose of warfarin. *Thromb Haemost* **2004**, *91*, 87-94.
30. Bodin, L.; Verstuyft, C.; Tregouet, D.A.; Robert, A.; Dubert, L.; Funck-Brentano, C.; Jaillon, P.; Beaune, P.; Laurent-Puig, P.; Becquemont, L.*, et al.* Cytochrome P450 2C9 (CYP2C9) and vitamin K epoxide reductase (VKORC1) genotypes as determinants of acenocoumarol sensitivity. *Blood* **2005**, *106*, 135-140.
31. Wadelius, M.; Chen, L.Y.; Lindh, J.D.; Eriksson, N.; Ghori, M.J.; Bumpstead, S.; Holm, L.; McGinnis, R.; Rane, A.; Deloukas, P. The largest prospective warfarin-treated cohort supports genetic forecasting. *Blood* **2009**, *113*, 784-792.
32. Schalekamp, T.; Brasse, B.P.; Roijers, J.F.; Chahid, Y.; van Geest-Daalderop, J.H.; de Vries-Goldschmeding, H.; van Wijk, E.M.; Egberts, A.C.; de Boer, A. VKORC1 and CYP2C9 genotypes and acenocoumarol anticoagulation status: interaction between both genotypes affects overanticoagulation. *Clinical pharmacology and therapeutics* **2006**, *80*, 13-22.
33. Schalekamp, T.; Brasse, B.P.; Roijers, J.F.; van Meegen, E.; van der Meer, F.J.; van Wijk, E.M.; Egberts, A.C.; de Boer, A. VKORC1 and CYP2C9 genotypes and phenprocoumon anticoagulation status: interaction between both genotypes affects dose requirement. *Clinical pharmacology and therapeutics* **2007**, *81*, 185-193.
34. Reitsma, P.H.; van der Heijden, J.F.; Groot, A.P.; Rosendaal, F.R.; Buller, H.R. A C1173T dimorphism in the VKORC1 gene determines coumarin sensitivity and bleeding risk. *PLoS Med* **2005**, *2*, e312.
35. Wadelius, M.; Chen, L.Y.; Downes, K.; Ghori, J.; Hunt, S.; Eriksson, N.; Wallerman, O.; Melhus, H.; Wadelius, C.; Bentley, D.*, et al.* Common VKORC1 and GGCX polymorphisms associated with warfarin dose. *Pharmacogenomics J* **2005**, *5*, 262-270.
36. D'Andrea, G.; D'Ambrosio, R.L.; Di Perna, P.; Chetta, M.; Santacroce, R.; Brancaccio, V.; Grandone, E.; Margaglione, M. A polymorphism in the VKORC1 gene is associated with an interindividual variability in the dose-anticoagulant effect of warfarin. *Blood* **2005**, *105*, 645-649.
37. Takeuchi, F.; McGinnis, R.; Bourgeois, S.; Barnes, C.; Eriksson, N.; Soranzo, N.; Whittaker, P.; Ranganath, V.; Kumanduri, V.; McLaren, W.*, et al.* A genome-wide association study confirms VKORC1, CYP2C9, and CYP4F2 as principal genetic determinants of warfarin dose. *PLoS genetics* **2009**, *5*, e1000433.
38. Caldwell, M.D.; Awad, T.; Johnson, J.A.; Gage, B.F.; Falkowski, M.; Gardina, P.; Hubbard, J.; Turpaz, Y.; Langaee, T.Y.; Eby, C.*, et al.* CYP4F2 genetic variant alters required warfarin dose. *Blood* **2008**, *111*, 4106-4112.

39. Schelleman, H.; Chen, J.; Chen, Z.; Christie, J.; Newcomb, C.W.; Brensinger, C.M.; Price, M.; Whitehead, A.S.; Kealey, C.; Thorn, C.F., *et al.* Dosing algorithms to predict warfarin maintenance dose in Caucasians and African Americans. *Clinical pharmacology and therapeutics* **2008**, *84*, 332-339.
40. International Warfarin Pharmacogenetics, C.; Klein, T.E.; Altman, R.B.; Eriksson, N.; Gage, B.F.; Kimmel, S.E.; Lee, M.T.; Limdi, N.A.; Page, D.; Roden, D.M., *et al.* Estimation of the warfarin dose with clinical and pharmacogenetic data. *The New England journal of medicine* **2009**, *360*, 753-764.
41. Perini, J.A.; Struchiner, C.J.; Silva-Assuncao, E.; Santana, I.S.; Rangel, F.; Ojopi, E.B.; Dias-Neto, E.; Suarez-Kurtz, G. Pharmacogenetics of warfarin: development of a dosing algorithm for brazilian patients. *Clinical pharmacology and therapeutics* **2008**, *84*, 722-728.
42. Sconce, E.A.; Khan, T.I.; Wynne, H.A.; Avery, P.; Monkhouse, L.; King, B.P.; Wood, P.; Kesteven, P.; Daly, A.K.; Kamali, F. The impact of CYP2C9 and VKORC1 genetic polymorphism and patient characteristics upon warfarin dose requirements: proposal for a new dosing regimen. *Blood* **2005**, *106*, 2329-2333.
43. Tham, L.S.; Goh, B.C.; Nafziger, A.; Guo, J.Y.; Wang, L.Z.; Soong, R.; Lee, S.C. A warfarin-dosing model in Asians that uses single-nucleotide polymorphisms in vitamin K epoxide reductase complex and cytochrome P450 2C9. *Clinical pharmacology and therapeutics* **2006**, *80*, 346-355.
44. Gage, B.F.; Eby, C.; Johnson, J.A.; Deych, E.; Rieder, M.J.; Ridker, P.M.; Milligan, P.E.; Grice, G.; Lenzini, P.; Rettie, A.E., *et al.* Use of pharmacogenetic and clinical factors to predict the therapeutic dose of warfarin. *Clinical pharmacology and therapeutics* **2008**, *84*, 326-331.
45. Eckman, M.H.; Rosand, J.; Greenberg, S.M.; Gage, B.F. Cost-effectiveness of using pharmacogenetic information in warfarin dosing for patients with nonvalvular atrial fibrillation. *Annals of internal medicine* **2009**, *150*, 73-83.
46. Schalekamp, T.; Boink, G.J.; Visser, L.E.; Stricker, B.H.; de Boer, A.; Klungel, O.H. CYP2C9 genotyping in acenocoumarol treatment: is it a cost-effective addition to international normalized ratio monitoring? *Clinical pharmacology and therapeutics* **2006**, *79*, 511-520.
47. Hughes, D.A.; Pirmohamed, M. Warfarin pharmacogenetics: economic considerations. *Pharmacoeconomics* **2007**, *25*, 899-902.
48. Le Tourneau, C.; Delord, J.P.; Goncalves, A.; Gavoille, C.; Dubot, C.; Isambert, N.; Campone, M.; Tredan, O.; Massiani, M.A.; Mauborgne, C., *et al.* Molecularly targeted therapy based on tumour molecular profiling versus conventional therapy for advanced cancer (SHIVA): a multicentre, open-label, proof-of-concept, randomised, controlled phase 2 trial. *Lancet Oncol* **2015**, *16*, 1324-1334.
49. Slamon, D.J.; Leyland-Jones, B.; Shak, S.; Fuchs, H.; Paton, V.; Bajamonde, A.; Fleming, T.; Eiermann, W.; Wolter, J.; Pegram, M., *et al.* Use of chemotherapy plus a monoclonal antibody against HER2 for metastatic breast cancer that overexpresses HER2. *The New England journal of medicine* **2001**, *344*, 783-792.
50. Lievre, A.; Bachet, J.B.; Boige, V.; Cayre, A.; Le Corre, D.; Buc, E.; Ychou, M.; Bouche, O.; Landi, B.; Louvet, C., *et al.* KRAS mutations as an independent prognostic factor in patients with advanced colorectal cancer treated with cetuximab. *Journal of clinical oncology : official journal of the American Society of Clinical Oncology* **2008**, *26*, 374-379.
51. Joensuu, H.; Roberts, P.J.; Sarlomo-Rikala, M.; Andersson, L.C.; Tervahartiala, P.; Tuveson, D.; Silberman, S.; Capdeville, R.; Dimitrijevic, S.; Druker, B., *et al.* Effect of the tyrosine kinase inhibitor STI571 in a patient with a metastatic gastrointestinal stromal tumor. *The New England journal of medicine* **2001**, *344*, 1052-1056.

52. DiMasi, J.A.; Grabowski, H.G. Economics of new oncology drug development. *Journal of clinical oncology : official journal of the American Society of Clinical Oncology* **2007**, *25*, 209-216.
53. Von Hoff, D.D. There are no bad anticancer agents, only bad clinical trial designs--twenty-first Richard and Hinda Rosenthal Foundation Award Lecture. *Clinical Cancer Research* **1998**, *4*, 1079-1086.
54. Thatcher, N.; Chang, A.; Parikh, P.; Rodrigues Pereira, J.; Ciuleanu, T.; von Pawel, J.; Thongprasert, S.; Tan, E.H.; Pemberton, K.; Archer, V., *et al.* Gefitinib plus best supportive care in previously treated patients with refractory advanced non-small-cell lung cancer: results from a randomised, placebo-controlled, multicentre study (Iressa Survival Evaluation in Lung Cancer). *Lancet* **2005**, *366*, 1527-1537.
55. Mok, T.S.; Wu, Y.L.; Thongprasert, S.; Yang, C.H.; Chu, D.T.; Saijo, N.; Sunpaweravong, P.; Han, B.; Margono, B.; Ichinose, Y., *et al.* Gefitinib or carboplatin-paclitaxel in pulmonary adenocarcinoma. *The New England journal of medicine* **2009**, *361*, 947-957.
56. Von Hoff, D.D.; Stephenson, J.J., Jr.; Rosen, P.; Loesch, D.M.; Borad, M.J.; Anthony, S.; Jameson, G.; Brown, S.; Cantafio, N.; Richards, D.A., *et al.* Pilot study using molecular profiling of patients' tumors to find potential targets and select treatments for their refractory cancers. *Journal of clinical oncology : official journal of the American Society of Clinical Oncology* **2010**, *28*, 4877-4883.
57. Doroshow, J.H. Selecting systemic cancer therapy one patient at a time: is there a role for molecular profiling of individual patients with advanced solid tumors? *Journal of clinical oncology : official journal of the American Society of Clinical Oncology* **2010**, *28*, 4869-4871.
58. Kim, E.S.; Herbst, R.S.; Wistuba, II; Lee, J.J.; Blumenschein, G.R., Jr.; Tsao, A.; Stewart, D.J.; Hicks, M.E.; Erasmus, J., Jr.; Gupta, S., *et al.* The BATTLE trial: personalizing therapy for lung cancer. *Cancer discovery* **2011**, *1*, 44-53.
59. Singh, K.; Peyser, B.; Trujillo, G.; Milazzo, N.; Savard, D.; Haga, S.B.; Musty, M.; Voora, D. Rationale and design of the SLCO1B1 genotype guided statin therapy trial. *Pharmacogenomics* **2016**, *17*, 1873-1880.
60. Mozaffarian, D.; Benjamin, E.J.; Go, A.S.; Arnett, D.K.; Blaha, M.J.; Cushman, M.; de Ferranti, S.; Després, J.-P.; Fullerton, H.J.; Howard, V.J., *et al.* Executive Summary: Heart Disease and Stroke Statistics—2015 Update. *Circulation* **2015**, *131*, 434-441.
61. Greenland, P.; Lauer, M.S. Cholesterol Lowering in 2015: Still Answering Questions About How and in Whom. *JAMA* **2015**, *314*, 127-128.
62. Cholesterol Treatment Trialists Collaborators. The effects of lowering LDL cholesterol with statin therapy in people at low risk of vascular disease: meta-analysis of individual data from 27 randomised trials. *The Lancet* **2012**, *380*, 581-590.
63. Cholesterol Treatment Trialists Collaborators. Efficacy and safety of LDL-lowering therapy among men and women: meta-analysis of individual data from 174 000 participants in 27 randomised trials. *The Lancet* **2015**, *385*, 1397-1405.
64. Taylor, F.; Huffman, M.D.; Macedo, A.F.; Moore, T.H.; Burke, M.; Davey Smith, G.; Ward, K.; Ebrahim, S. Statins for the primary prevention of cardiovascular disease. *The Cochrane database of systematic reviews* **2013**, CD004816.
65. Stone, N.J.; Robinson, J.G.; Lichtenstein, A.H.; Bairey Merz, C.N.; Blum, C.B.; Eckel, R.H.; Goldberg, A.C.; Gordon, D.; Levy, D.; Lloyd-Jones, D.M., *et al.* 2013 ACC/AHA Guideline on the Treatment of Blood Cholesterol to Reduce Atherosclerotic Cardiovascular Risk in Adults. *Journal of the American College of Cardiology* **2014**, *63*, 2889-2934.
66. Pencina, M.J.; Navar-Boggan, A.M.; D'Agostino, R.B., Sr.; Williams, K.; Neely, B.; Sniderman, A.D.; Peterson, E.D. Application of new cholesterol guidelines to a population-based sample. *The New England journal of medicine* **2014**, *370*, 1422-1431.

67. Hirsh, B.J.; Smilowitz, N.R.; Rosenson, R.S.; Fuster, V.; Sperling, L.S. Utilization of and Adherence to Guideline-Recommended Lipid-Lowering Therapy After Acute Coronary Syndrome: Opportunities for Improvement. *J Am Coll Cardiol* **2015**, *66*, 184-192.
68. Bermingham, M.; Hayden, J.; Dawkins, I.; Miwa, S.; Gibson, D.; McDonald, K.; Ledwidge, M. Prospective analysis of LDL-C goal achievement and self-reported medication adherence among statin users in primary care. *Clin Ther* **2011**, *33*, 1180-1189.
69. Ho, P.M.; Magid, D.J.; Shetterly, S.M.; Olson, K.L.; Maddox, T.M.; Peterson, P.N.; Masoudi, F.A.; Rumsfeld, J.S. Medication nonadherence is associated with a broad range of adverse outcomes in patients with coronary artery disease. *Am Heart J* **2008**, *155*, 772-779.
70. Vodonos, A.; Ostapenko, I.; Toledano, R.; Henkin, Y.; Zahger, D.; Wolak, T.; Sherf, M.; Novack, V. Statin adherence and LDL cholesterol levels. Should we assess adherence prior to statin upgrade? *European journal of internal medicine* **2015**, *26*, 268-272.
71. Franklin, J.M.; Krumme, A.A.; Tong, A.Y.; Shrank, W.H.; Matlin, O.S.; Brennan, T.A.; Choudhry, N.K. Association between trajectories of statin adherence and subsequent cardiovascular events. *Pharmacoepidemiol Drug Saf* **2015**, *24*, 1105-1113.
72. De Vera, M.A.; Bhole, V.; Burns, L.C.; Lacaille, D. Impact of statin adherence on cardiovascular disease and mortality outcomes: a systematic review. *British journal of clinical pharmacology* **2014**, *78*, 684-698.
73. Osterberg, L.; Blaschke, T. Adherence to medication. *The New England journal of medicine* **2005**, *353*, 487-497.
74. Fung, V.; Sinclair, F.; Wang, H.; Dailey, D.; Hsu, J.; Shaber, R. Patients' perspectives on nonadherence to statin therapy: a focus-group study. *Perm J* **2010**, *14*, 4-10.
75. Cohen, J.D.; Brinton, E.A.; Ito, M.K.; Jacobson, T.A. Understanding Statin Use in America and Gaps in Patient Education (USAGE): an internet-based survey of 10,138 current and former statin users. *J Clin Lipidol* **2012**, *6*, 208-215.
76. Ong, F.S.; Deignan, J.L.; Kuo, J.Z.; Bernstein, K.E.; Rotter, J.I.; Grody, W.W.; Das, K. Clinical utility of pharmacogenetic biomarkers in cardiovascular therapeutics: a challenge for clinical implementation. *Pharmacogenomics* **2012**, *13*, 465-475.
77. Pasternak, R.C.; Smith, S.C.; Bairey-Merz, C.N.; Grundy, S.M.; Cleeman, J.I.; Lenfant, C. ACC/AHA/NHLBI clinical advisory on the use and safety of statins. *Journal of the American College of Cardiology* **2002**, *40*, 567-572.
78. Thompson, P.D.; Clarkson, P.M.; Rosenson, R.S.; National Lipid Association Statin Safety Task Force Muscle Safety Expert, P. An assessment of statin safety by muscle experts. *Am J Cardiol* **2006**, *97*, 69C-76C.
79. Alfirevic, A.; Neely, D.; Armitage, J.; Chinoy, H.; Cooper, R.G.; Laaksonen, R.; Carr, D.F.; Bloch, K.M.; Fahy, J.; Hanson, A.*, et al.* Phenotype standardization for statin-induced myotoxicity. *Clinical pharmacology and therapeutics* **2014**, *96*, 470-476.
80. Patel, J.; Superko, H.R.; Martin, S.S.; Blumenthal, R.S.; Christopher-Stine, L. Genetic and immunologic susceptibility to statin-related myopathy. *Atherosclerosis* **2015**, *240*, 260-271.
81. Stroes, E.S.; Thompson, P.D.; Corsini, A.; Vladutiu, G.D.; Raal, F.J.; Ray, K.K.; Roden, M.; Stein, E.; Tokgozoglu, L.; Nordestgaard, B.G.*, et al.* Statin-associated muscle symptoms: impact on statin therapy-European Atherosclerosis Society Consensus Panel Statement on Assessment, Aetiology and Management. *Eur Heart J* **2015**, *36*, 1012-1022.
82. Search Collaborative Group; Link, E.; Parish, S.; Armitage, J.; Bowman, L.; Heath, S.; Matsuda, F.; Gut, I.; Lathrop, M.; Collins, R. SLCO1B1 variants and statin-induced myopathy--a genomewide study. *The New England journal of medicine* **2008**, *359*, 789-799.
83. Hou, Q.; Li, S.; Li, L.; Li, Y.; Sun, X.; Tian, H. Association Between SLCO1B1 Gene T521C Polymorphism and Statin-Related Myopathy Risk: A Meta-Analysis of Case-Control Studies. *Medicine* **2015**, *94*, e1268.

84. Pasanen, M.K.; Backman, J.T.; Neuvonen, P.J.; Niemi, M. Frequencies of single nucleotide polymorphisms and haplotypes of organic anion transporting polypeptide 1B1 SLCO1B1 gene in a Finnish population. *Eur J Clin Pharmacol* **2006**, *62*, 409-415.
85. Kimoto, E.; Yoshida, K.; Balogh, L.M.; Bi, Y.A.; Maeda, K.; El-Kattan, A.; Sugiyama, Y.; Lai, Y. Characterization of organic anion transporting polypeptide (OATP) expression and its functional contribution to the uptake of substrates in human hepatocytes. *Mol Pharm* **2012**, *9*, 3535-3542.
86. Voora, D.; Shah, S.H.; Spasojevic, I.; Ali, S.; Reed, C.R.; Salisbury, B.A.; Ginsburg, G.S. The SLCO1B1*5 genetic variant is associated with statin-induced side effects. *J Am Coll Cardiol* **2009**, *54*, 1609-1616.
87. Wilke, R.A.; Ramsey, L.B.; Johnson, S.G.; Maxwell, W.D.; McLeod, H.L.; Voora, D.; Krauss, R.M.; Roden, D.M.; Feng, Q.; Cooper-Dehoff, R.M.*, et al.* The clinical pharmacogenomics implementation consortium: CPIC guideline for SLCO1B1 and simvastatin-induced myopathy. *Clinical pharmacology and therapeutics* **2012**, *92*, 112-117.
88. Birmingham, B.K.; Bujac, S.R.; Elsby, R.; Azumaya, C.T.; Wei, C.; Chen, Y.; Mosqueda-Garcia, R.; Ambrose, H.J. Impact of ABCG2 and SLCO1B1 polymorphisms on pharmacokinetics of rosuvastatin, atorvastatin and simvastatin acid in Caucasian and Asian subjects: a class effect? *Eur J Clin Pharmacol* **2015**, *71*, 341-355.
89. de Keyser, C.E.; Peters, B.J.; Becker, M.L.; Visser, L.E.; Uitterlinden, A.G.; Klungel, O.H.; Verstuyft, C.; Hofman, A.; Maitland-van der Zee, A.-H.; Stricker, B.H. The SLCO1B1 c. 521T> C polymorphism is associated with dose decrease or switching during statin therapy in the Rotterdam Study. *Pharmacogenetics and genomics* **2014**, *24*, 43-51.
90. Danik, J.S.; Chasman, D.I.; MacFadyen, J.G.; Nyberg, F.; Barratt, B.J.; Ridker, P.M. Lack of association between SLCO1B1 polymorphisms and clinical myalgia following rosuvastatin therapy. *Am Heart J* **2013**, *165*, 1008-1014.
91. Martin, N.G.; Li, K.W.; Murray, H.; Putt, W.; Packard, C.J.; Humphries, S.E. The effects of a single nucleotide polymorphism in SLCO1B1 on the pharmacodynamics of pravastatin. *British journal of clinical pharmacology* **2012**, *73*, 303-306.
92. Niemi, M.; Pasanen, M.K.; Neuvonen, P.J. Organic anion transporting polypeptide 1B1: a genetically polymorphic transporter of major importance for hepatic drug uptake. *Pharmacol Rev* **2011**, *63*, 157-181.
93. Ramsey, L.B.; Johnson, S.G.; Caudle, K.E.; Haidar, C.E.; Voora, D.; Wilke, R.A.; Maxwell, W.D.; McLeod, H.L.; Krauss, R.M.; Roden, D.M.*, et al.* The clinical pharmacogenetics implementation consortium guideline for SLCO1B1 and simvastatin-induced myopathy: 2014 update. *Clinical pharmacology and therapeutics* **2014**, *96*, 423-428.
94. Voora, D.; Ginsburg, G.S. Clinical application of cardiovascular pharmacogenetics. *J Am Coll Cardiol* **2012**, *60*, 9-20.
95. Donnelly, L.A.; Doney, A.S.; Tavendale, R.; Lang, C.C.; Pearson, E.R.; Colhoun, H.M.; McCarthy, M.I.; Hattersley, A.T.; Morris, A.D.; Palmer, C.N. Common nonsynonymous substitutions in SLCO1B1 predispose to statin intolerance in routinely treated individuals with type 2 diabetes: a go-DARTS study. *Clinical pharmacology and therapeutics* **2011**, *89*, 210-216.
96. Li, J.H.; Joy, S.V.; Haga, S.B.; Orlando, L.A.; Kraus, W.E.; Ginsburg, G.S.; Voora, D. Genetically guided statin therapy on statin perceptions, adherence, and cholesterol lowering: a pilot implementation study in primary care patients. *J Pers Med* **2014**, *4*, 147-162.
97. Mosley, S.A.; Hicks, J.K.; Portman, D.G.; Donovan, K.A.; Gopalan, P.; Schmit, J.; Starr, J.; Silver, N.; Gong, Y.; Langaee, T.*, et al.* Design and rational for the precision medicine guided treatment for cancer pain pragmatic clinical trial. *Contemp Clin Trials* **2018**, *68*, 7-13.

98. P. D. Q. Supportive Palliative Care Editorial Board. Cancer Pain (PDQ(R)): Health Professional Version. In *PDQ Cancer Information Summaries*, National Cancer Institute (US): Bethesda (MD), 2002.
99. National Comprehensive Cancer Network. *NCCN clinical practice guidelines in oncology: Adult cancer pain. Version 2.2016*; https://www.nccn.org, 2016.
100. Temel, J.S.; Greer, J.A.; Muzikansky, A.; Gallagher, E.R.; Admane, S.; Jackson, V.A.; Dahlin, C.M.; Blinderman, C.D.; Jacobsen, J.; Pirl, W.F.*, et al.* Early palliative care for patients with metastatic non-small-cell lung cancer. *The New England journal of medicine* **2010**, *363*, 733-742.
101. Caraceni, A.; Hanks, G.; Kaasa, S.; Bennett, M.I.; Brunelli, C.; Cherny, N.; Dale, O.; De Conno, F.; Fallon, M.; Hanna, M.*, et al.* Use of opioid analgesics in the treatment of cancer pain: evidence-based recommendations from the EAPC. *Lancet Oncol* **2012**, *13*, e58-68.
102. Fine, P.G.; Portenoy, R.K.; Ad Hoc Expert Panel on Evidence, R.; Guidelines for Opioid, R. Establishing "best practices" for opioid rotation: conclusions of an expert panel. *J Pain Symptom Manage* **2009**, *38*, 418-425.
103. Zhao, F.; Chang, V.T.; Cleeland, C.; Cleary, J.F.; Mitchell, E.P.; Wagner, L.I.; Fisch, M.J. Determinants of pain severity changes in ambulatory patients with cancer: an analysis from Eastern Cooperative Oncology Group trial E2Z02. *Journal of clinical oncology : official journal of the American Society of Clinical Oncology* **2014**, *32*, 312-319.
104. Angst, M.S.; Phillips, N.G.; Drover, D.R.; Tingle, M.; Ray, A.; Swan, G.E.; Lazzeroni, L.C.; Clark, J.D. Pain sensitivity and opioid analgesia: a pharmacogenomic twin study. *Pain* **2012**, *153*, 1397-1409.
105. Fillingim, R.B.; Wallace, M.R.; Herbstman, D.M.; Ribeiro-Dasilva, M.; Staud, R. Genetic contributions to pain: a review of findings in humans. *Oral Dis* **2008**, *14*, 673-682.
106. Gan, S.H.; Ismail, R.; Wan Adnan, W.A.; Zulmi, W. Impact of CYP2D6 genetic polymorphism on tramadol pharmacokinetics and pharmacodynamics. *Mol Diagn Ther* **2007**, *11*, 171-181.
107. Susce, M.T.; Murray-Carmichael, E.; de Leon, J. Response to hydrocodone, codeine and oxycodone in a CYP2D6 poor metabolizer. *Prog Neuropsychopharmacol Biol Psychiatry* **2006**, *30*, 1356-1358.
108. Crews, K.R.; Gaedigk, A.; Dunnenberger, H.M.; Leeder, J.S.; Klein, T.E.; Caudle, K.E.; Haidar, C.E.; Shen, D.D.; Callaghan, J.T.; Sadhasivam, S.*, et al.* Clinical Pharmacogenetics Implementation Consortium guidelines for cytochrome P450 2D6 genotype and codeine therapy: 2014 update. *Clinical pharmacology and therapeutics* **2014**, *95*, 376-382.
109. Baber, M.; Chaudhry, S.; Kelly, L.; Ross, C.; Carleton, B.; Berger, H.; Koren, G. The pharmacogenetics of codeine pain relief in the postpartum period. *Pharmacogenomics J* **2015**, *15*, 430-435.
110. Ciszkowski, C.; Madadi, P.; Phillips, M.S.; Lauwers, A.E.; Koren, G. Codeine, ultrarapid-metabolism genotype, and postoperative death. *The New England journal of medicine* **2009**, *361*, 827-828.
111. Gasche, Y.; Daali, Y.; Fathi, M.; Chiappe, A.; Cottini, S.; Dayer, P.; Desmeules, J. Codeine intoxication associated with ultrarapid CYP2D6 metabolism. *The New England journal of medicine* **2004**, *351*, 2827-2831.
112. Eckhardt, K.; Li, S.; Ammon, S.; Schanzle, G.; Mikus, G.; Eichelbaum, M. Same incidence of adverse drug events after codeine administration irrespective of the genetically determined differences in morphine formation. *Pain* **1998**, *76*, 27-33.
113. Lotsch, J.; Rohrbacher, M.; Schmidt, H.; Doehring, A.; Brockmoller, J.; Geisslinger, G. Can extremely low or high morphine formation from codeine be predicted prior to therapy initiation? *Pain* **2009**, *144*, 119-124.

114. Andreassen, T.N.; Eftedal, I.; Klepstad, P.; Davies, A.; Bjordal, K.; Lundstrom, S.; Kaasa, S.; Dale, O. Do CYP2D6 genotypes reflect oxycodone requirements for cancer patients treated for cancer pain? A cross-sectional multicentre study. *Eur J Clin Pharmacol* **2012**, *68*, 55-64.
115. Samer, C.F.; Daali, Y.; Wagner, M.; Hopfgartner, G.; Eap, C.B.; Rebsamen, M.C.; Rossier, M.F.; Hochstrasser, D.; Dayer, P.; Desmeules, J.A. The effects of CYP2D6 and CYP3A activities on the pharmacokinetics of immediate release oxycodone. *Br J Pharmacol* **2010**, *160*, 907-918.
116. Zwisler, S.T.; Enggaard, T.P.; Noehr-Jensen, L.; Pedersen, R.S.; Mikkelsen, S.; Nielsen, F.; Brosen, K.; Sindrup, S.H. The hypoalgesic effect of oxycodone in human experimental pain models in relation to the CYP2D6 oxidation polymorphism. *Basic Clin Pharmacol Toxicol* **2009**, *104*, 335-344.
117. Zwisler, S.T.; Enggaard, T.P.; Mikkelsen, S.; Brosen, K.; Sindrup, S.H. Impact of the CYP2D6 genotype on post-operative intravenous oxycodone analgesia. *Acta Anaesthesiol Scand* **2010**, *54*, 232-240.
